# Assessment of Radiation Induced Therapeutic Effect and Cytotoxicity in Cancer Patients Based on Transcriptomic Profiling

**DOI:** 10.3390/ijms17020250

**Published:** 2016-02-19

**Authors:** Sajjad Karim, Zeenat Mirza, Adeel G. Chaudhary, Adel M. Abuzenadah, Mamdooh Gari, Mohammed H. Al-Qahtani

**Affiliations:** 1 Center of Excellence in Genomic Medicine Research, Faculty of Applied Medical Sciences, King Abdulaziz University, Jeddah 21589, Saudi Arabia; skarim1@kau.edu.sa (S.K.); adeel.gc@gmail.com (A.G.C.); adel_abuzenadah@hotmail.com (A.M.A.); mgari@kau.edu.sa (M.G.); mhalqahtani@kau.edu.sa (M.H.A.); 2 King Fahd Medical Research Center, King Abdulaziz University, P.O. Box 80216, Jeddah 21589, Saudi Arabia; 3 King Abdullah City for Science and Technology Innovation Center for Personalized Medicine, King Abdulaziz University, P.O. Box 80216, Jeddah 21589, Saudi Arabia

**Keywords:** radiation therapy, toxicity, microarray, cancer, pathway analysis

## Abstract

Toxicity induced by radiation therapy is a curse for cancer patients undergoing treatment. It is imperative to understand and define an ideal condition where the positive effects notably outweigh the negative. We used a microarray meta-analysis approach to measure global gene-expression before and after radiation exposure. Bioinformatic tools were used for pathways, network, gene ontology and toxicity related studies. We found 429 differentially expressed genes at fold change >2 and *p*-value <0.05. The most significantly upregulated genes were synuclein alpha (*SNCA*), carbonic anhydrase I (*CA1*), X-linked Kx blood group (*XK*), glycophorin A and B (*GYPA* and *GYPB*), and hemogen (*HEMGN)*, while downregulated ones were membrane-spanning 4-domains, subfamily A member 1 (*MS4A1*), immunoglobulin heavy constant mu (*IGHM*), chemokine (C-C motif) receptor 7 (*CCR7*), BTB and CNC homology 1 transcription factor 2 (*BACH2*), and B-cell CLL/lymphoma 11B (*BCL11B*). Pathway analysis revealed calcium-induced T lymphocyte apoptosis and the role of nuclear factor of activated T-cells (*NFAT*) in regulation of the immune response as the most inhibited pathways, while apoptosis signaling was significantly activated. Most of the normal biofunctions were significantly decreased while cell death and survival process were activated. Gene ontology enrichment analysis revealed the immune system process as the most overrepresented group under the biological process category. Toxicity function analysis identified liver, kidney and heart to be the most affected organs during and after radiation therapy. The identified biomarkers and alterations in molecular pathways induced by radiation therapy should be further investigated to reduce the cytotoxicity and development of fatigue.

## 1. Introduction

Regardless of its potential hazards, curative benefits of radiation have been reported in medicine, oncology in particular. It is routinely used in cancer treatment prior to surgery to help shrink the tumor, as a palliative therapy to relieve pain, pressure and other neurologic or obstructive symptoms, and post-surgery to kill any remaining cancer cells and to prevent tumor recurrence or in synergy with chemotherapy [1]. The three prime categories of radiation therapy (RT) based on the differences relating to the position of the radiation sources are external beam RT (teletherapy) where the radiation source is outside the body, sealed source RT (brachytherapy) employs sealed radioactive sources placed permanently or temporarily precisely in the area under treatment, and systemic unsealed source radioisotope therapy is given by oral ingestion of radioisotopes.

Localized RT, using ionizing radiation, is the most common therapeutic option recommended for the treatment of ~60% of non-metastatic cancer patients [2,3,4]. RT has led to high cure and improved survival rates; however, this is mitigated by the associated toxicities that decrease the quality of life for survivors as ~10% of patients suffer significant toxicity because of adverse side effects [5]. Additional risk factors include age, chemotherapy, anatomical variations and coexisting illnesses, for instance, diabetes and autoimmune diseases [6,7,8]. Extra caution and personalized or tailored palliative radiotherapy administration is essential for patients in the end-stage of life with terminal cancer [9].

Several *ex vivo* efforts to correlate radiation toxicity with cellular responses have been done. Studies have reported decreased survival of cultured skin fibroblasts [10], paradoxical decrease in radiation-induced apoptosis [11], and development of abnormal numbers of chromosome aberrations [12] in patients after radiation exposure. Still, the etiology of fatigue and severity of RT associated side effects during cancer treatment are not well understood. Some studies have correlated certain genes, pathways and molecular processes to fatigue [13,14]. It has been well demonstrated that irradiated dying cancer cells release tumor antigens. The extracellular antigens and dying tumor cells are engulfed by circulating bone marrow-derived antigen-presenting cells (APCs). Subsequent to antigen uptake, APCs move to lymph nodes, where they engage with helper T cells for post-stimulation and APC activation, and further stimulate the tumor specific cytotoxic T lymphocytes that could potentially clear tumor cells both at primary and metastatic sites [15]. Radiation-induced immune-modulation happens in two phases. First, radiation induces endogenous damage-associated molecular pattern (DAMP) molecules also known as alarmins and may include intracellular proteins, like heat-shock proteins, high-mobility group box 1 (HMGB1) and proinflammatory S100 proteins linked to inflammation and cancer [16] or non-protein molecules like ATP [17], uric acid [18], heparin sulfate and DNA [19]. The release of DAMP molecules are considered as “danger signals” and trigger stimulation and maturation of dendritic cells which can then present foreign antigens and cause stimulation of T lymphocytes. In this event, radiation normalizes tumor vasculature, increases tumor cells’ immune recognition and modulates the tumor cell phenotype. Radiation treatment can cause upregulation of chemokines and adhesion molecules, providing signals for T cells to be attracted to the tumor; and upregulation of Major Histocompatibility Complex molecules and tumor-associated antigens, making it easier for endogenous or immunotherapy-induced T cells to recognize and kill tumors via immunogenic modulation [20]. Second, amplification by abrogating immune checkpoint factors with simultaneous co-stimulation of effector factors can ultimately lead to the induction of multiple unique T-cell populations (antigen cascade) that can kill antigen disparate tumor cells at metastatic sites (systemic effect). It has been show that *HMGB1*, a nuclear non-histone chromatin-binding protein, is secreted at the late stages of cellular death and is also secreted by apoptotic tumor cells after chemotherapy or radiotherapy promoting antitumor responses [21,22].

We hypothesized that radiation induced toxicity in affected cells respond to DNA damage and have an abnormal transcriptional profile leading to alterations in key pathways. To comprehend the underlying mechanisms of radiation induced response, we adopted transcriptomics based molecular assessment of induced features during cancer treatment.

## 2. Results

The prime focus of this study was to determine the beneficial and deleterious effects of RT using transcriptional profiling and pathway prediction models.

### 2.1. Identification of Differentially Expressed Genes

**Figure 1:**
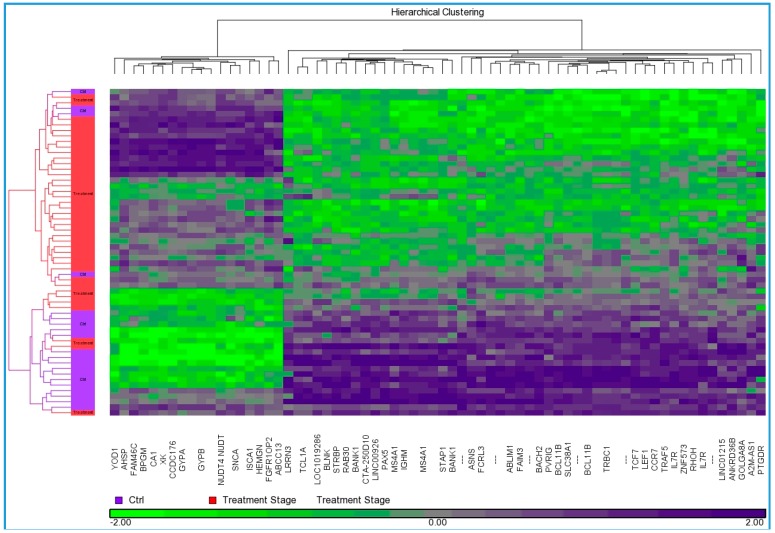
Agglomerative average-linkage hierarchical clustering for differentially expressed genes between radiation treatment stage and controls. Dendrogram obtained using Partek GS 6.6 software shows the change in expression levels of genes (*n* = 429, 32-up and 397-downregulated) in RT treated cancer patients compared to untreated controls, Differentially Expressed Genes (DEGs) on *X* axis and treatment stage on *Y* axis. The cluster color represents the normalized expression level of a given gene in response to radiation treatment, Purple denotes upregulation and green denotes downregulation according the color scale.

We found 429 differentially expressed genes, 32 were up while the remaining 397 genes were downregulated, which were used to understand the molecular mechanism and processes involved in radiation therapy (Supplementary Table S1, Table 1, Figure 1). The most significantly upregulated genes were alpha synuclein (*SNCA*), carbonic anhydrase I (*CA1*), X-linked Kx blood group (*XK*), glycophorin A, and B (*GYPA* and *GYPB*), hemogen (*HEMGN*), 2,3-bisphosphoglycerate mutase (*BPGM*), ATP-binding cassette sub-family C member 13 (*ABCC13*), and ferrochelatase (*FECH*), while most downregulated genes were membrane-spanning 4-domains, subfamily A member 1 (*MS4A1*), immunoglobulin heavy constant mu (*IGHM*), chemokine (C-C motif) receptor 7 (*CCR7*), BTB and CNC homology 1 transcription factor 2 (*BACH2*), B-cell CLL/lymphoma 11B (*BCL11B*), paired box 5 (*PAX5*), lymphoid enhancer-binding factor 1 (*LEF1*), Fas apoptotic inhibitory molecule 3 (*FAIM3*), A2M antisense RNA 1 (*A2M-AS1*) and TNF receptor-associated factor 5 (*TRAF5*).

### 2.2. Pathways and Networks Underlying Immune Dysfunction

**Figure 2:**
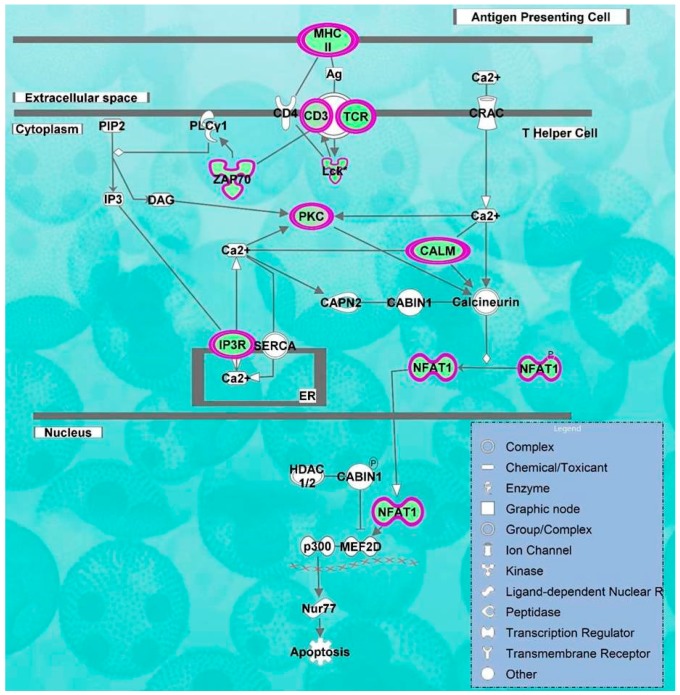
Inhibition of Calcium-induced T Lymphocyte Apoptosis pathway. Based on overlap of identified DEGs to Calcium-induced T Lymphocyte Apoptosis pathways, IPA had predicted its inhibition. *CD3*, *CAMK4*, *TRGV9*, *NFAT*, *ZAP70*, *LCK*, *PRKC*, *HLA-DOB*, *ITPR1* genes involved in this pathway were downregulated as shown by the purple circles. XXXX line indicate DNA strand. The white end arrow means “translocation” and the dark end arrow means “acts on”.

**Figure 3:**
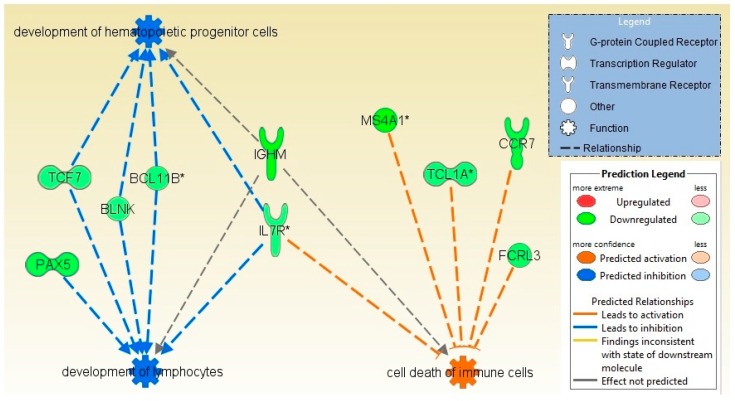
Functional analysis and regulatory effect of differentially expressed genes. Using Ingenuity Pathways Analysis (IPA), Differentially Expressed Genes (DEGs) were overlaid on to the network to find a biological regulatory functional effect based on previously reported interactions in the literature. Function colored with blue denote inhibition and orange denotes activation; development of hematopoietic progenitor cells and development of lymphocytes were inhibited whereas cell death of immune cells as activated function.

Molecular pathway analysis of radiation treatment associated genes has predicted biofunctions and molecular networks which may be involved in the fatigue and cytotoxicity. Biofunctions including cell-mediated immune response, cell signaling, mineral metabolism, cellular function and maintenance were predicted to be significantly decreased while cell death and survival process were significantly activated (*p*-value = 1.76 × 10^−7^). Transcriptomic signatures displayed noteworthy disturbances in signaling pathways like calcium-induced T lymphocyte apoptosis (z-score = −3.31, Figure 2), and role of NFAT in regulation of the immune response (z-score = −3.77) were inhibited while apoptosis signaling (z-score = 2.23) and cytotoxic T lymphocyte-mediated apoptosis of target cells (z-score = 1.342) were predicted to be activated (Table 2). Cell death and survival process was predicted to be significantly increased/activated (cell death of immune cells, *p*-value = 1.76 × 10^−7^, z-score = 2.209; cell death of lymphocytes, *p*-value = 3.84 × 10^−5^, z-score = 2.017; apoptosis of leukocytes, *p*-value = 5.72 × 10^−4^, z-score = 2.424 and apoptosis of hematopoietic cell lines, *p*-value = 3.16 × 10^−3^, z-score = 2.489) (Figure 3). However, most of biofunctions were predicted to be decreased like cell-mediated immune response (T cell development, -homeostasis and -migration, *p*-value = 8.45 × 10^−11^, z-score = −2.867), cell signaling, and mineral metabolism (accumulation of Ca^2+^, mobilization of Ca^2+^, *p*-value = 5.72 × 10^−4^, z-score = −2.764), cellular function and maintenance (homeostasis of leukocytes, cellular homeostasis, *p*-value = 3.88 × 10^−11^, z-score = −3.122) (Supplementary Table S2).

**Figure 4:**
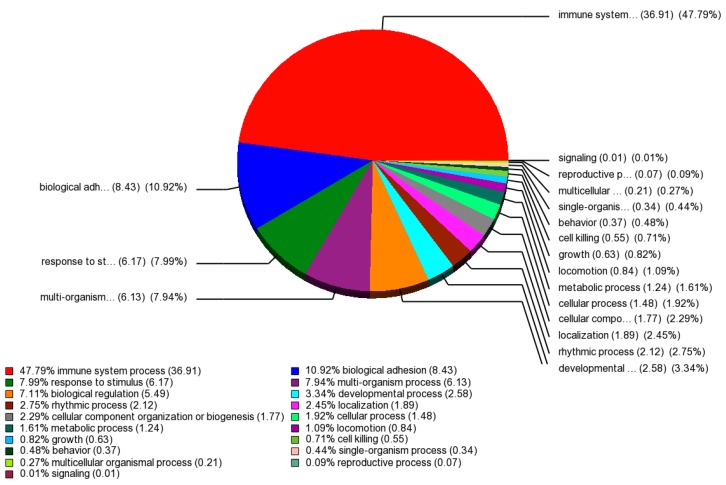
Gene Ontology of differentially expressed genes. Pie Chart obtained using Partek GS 6.6 software shows the change in the biological process of the immune system, biological adhesion, response to stimulus, multi-organism process, biological regulation and developmental process as a significantly affected process.

A Gene Ontology (GO) enrichment analysis was performed on those 429 differentially expressed probe sets that intersect on the basis of a *p*-value <0.05 and FC >2 between baseline and endpoint treatment groups. (Supplementary Table S3). The GO enrichment diagram illustrates functional groups that are significantly overrepresented in different categories (Figure 4). The most significantly overrepresented groups in the categories cellular component, molecular function, and biological process were “cell part”, “nucleic acid binding transcription factor activity”, and “immune system process”, respectively.

### 2.3. Toxicity Function Analysis

IPA based toxicity function analysis predicted liver, kidney and heart to be most affected organs during and after radiation therapy (Table 3). Alteration in *CCR7*, *IMPDH2*, *IL6ST*, *MYC*, *LPIN1*, *PDE7A* and *RORA* was significantly associated with hepatotoxicity including liver damage, -hyperplasia, -inflammation, -steatosis, -fibrosis, -necrosis and -proliferation. Altered expression of *AQP3*, *AAK1*, *BCL2*, *BIRC3*, *BNIP3*, *DDX17*, *FOXO1*, *ITPR1*, *MYC*, *PRKCA*, *SNCA*, *TNFRSF25*, *CCR7*, *MS4A1* and *IMPDH2* was responsible for renal necrosis, nephrosis, nephritis, proliferation and kidney failure. Cardiotoxicity (cardiac proliferation, -arteriopathy, -necrosis, -infarction and heart failure) was found to be significantly associated with the following altered genes: *CA1*, *FOXP1*, *NOG*, *ABCG1*, *CD47*, *DOCK9*, *MARCH6*, *PDE7A*, *PRKCH BNIP3* and *MIAT* during radiation therapy.

## 3. Discussion

Up to 90% of cancer patients treated with radiation experience cumulative fatigue that is pervasive and affects quality of life [23]. Cancer treatment elicits inflammatory and stress response, mitochondrial function impairment, endocrine dysfunction and immune dysregulation. This induces a cascade of biological changes which get translated into cancer-related fatigue (CRF) manifested with alteration in skeletal muscle function contributing to physical disability along with cognitive and behavioral symptoms. The above-mentioned fatigue has a multifactorial etiology and is reported to be associated with neuroendocrine, metabolic, immune/inflammatory, and genetic biomarkers whose systemic identification will help to understand its etiology and develop better therapies to lessen CRF burden [24]. Though, fatigue felt by the patients might also be due to conditions like anaemia, thyroid dysfunction, pain, depression and sleep deprivation [25].

In our results, α-synuclein (SNCA) (*p*-value = 2.70 × 10^−6^ and fold change = 3.5) was one of the differentially expressed gene identified in the microarray meta-analysis, which was validated [5] and reported to be associated with neuroinflammation [26]. SNCA is primarily involved in diseases like Parkinson’s and hereditary amyloidosis. During localized radiation therapy, its upregulation activates inflammatory pathways and signals the associated cancer-related fatigue [5]. We also found upregulation of carbonic anhydrase I (CA1) in our results which is in accordance with the previously published reports [5]. CA1 is a cytosolic protein and belongs to the large family of zinc metalloenzymes. It is found at the highest level in erythrocytes. It functions by catalyzing the reversible hydration of carbon dioxide and participates in a variety of biological processes like respiration, maintenance of acid–base balance, bone resorption and calcification [27].

One of the most down regulated genes in our study was membrane-spanning 4-domains, subfamily A, member 1 (MS4A1). It encodes for CD20 protein, a B1 cell-surface antigen of human B lymphocytes and plays a role in hematopoietic cell activation [28]. Lower *MS4A1* gene expression levels will possibly contribute to the lower CD20 protein expression [29]. Association of MS4A1 down regulation and fatigue intensification has been recently reported by Hsiao *et al.*, suggesting that fatigue during RT may be related to impairment in B-cell immune response [30]. MS4A1 was included in molecular signatures that reflects a response to radiation in mice and humans [31]. Reportedly, MS4A1 has indirect interaction with *CA1* and *SNCA* genes associated with cellular movement and immune cell trafficking [30]. Our gene ontology enrichment analysis revealed immune system process as the most significantly overrepresented group under biological process category that has been reported to be positively associated with CRF [32].

We found that apoptosis signaling and cytotoxic T lymphocyte-mediated apoptosis of target cells pathways were strongly associated with RT. Radiation induced stress activates p53-dependent apoptosis marked by increased formation of intracellular reactive oxygen species (ROS) and activation of stress responsive pathways like p38MAPK [33]. Radiation acts as an apoptotic stimuli and cause changes in the inner mitochondrial membrane permeability [34]. This leads to release of regulatory proteins such as cytochrome c which binds and activates Apaf-1, forming an “apoptosome” [35,36]. ATP activates apoptosome complex, which thereby activates procaspase-9. Smac/DIABLO and the serine protease, and HtrA2/Omi promote apoptosis by inhibiting IAP (inhibitors of apoptosis proteins) activity. During apoptosis, the mitochondria releases pro-apoptotic proteins like AIF (apoptosis-inducing factor), endonuclease G and CAD (caspase-activated DNase) [34].

Toxicity functional analysis revealed that radiation therapy does carry a huge price, *i.e.*, at the cost of hepato- , cardio-, and nephrotoxicity. Cases of radiation-induced liver diseases are becoming frequent. Radioembolization may affect the normal liver parenchyma and produce pertinent toxic effects like cholecystitis, gastrointestinal ulceration, pneumonitis, and liver toxicity as a result of radiation of non-target organs [37]. Long-term radiation induced damage in different tissues possibly is a consequence of injury to microvascular endothelial cells causing their apoptosis. Irradiation causes oxidative damage to DNA (both mitochondrial and nuclear) and significantly depletes mitochondrial glutathione which further enhances *in vitro* as well as *in vivo* toxicity levels [38].

Accumulating evidences indicate that radiotherapy involving the heart can result in premature ischemic heart disease in cancer survivors especially in those who had concurrently received chemotherapy specifically with doxorubicin (adriamycin, a drug that can cause heart muscle damage). Cardiac complication and damage can manifest even years after high-dose radiation treatment [39]. Severe RT induced coronary artery disease complications include pericarditis, myocardial fibrosis (scarring), stenosis (narrowing), angina and extensive blockage, valvular injury and myocardial infarction [40,41]. The relative risk of death from a fatal myocardial infarction increased from 1.5 to 3.0 times in patients who have received mediastinal RT as compared to those who have not [42].

Radiation induced nephritis is a degenerative inflammatory disease affecting kidneys after exposure to radioactive substances or body irradiation. Administration of radiometal-labelled peptide conjugates or combined high dose chemotherapy and RT in stem cell transplantation reportedly increases nephrotoxicity [43]. Radiation exposure causes renal endothelial damage and other kidney diseases and so renal shielding during total body irradiation is perhaps protective [44].

There are some factors accounting for rare adverse radiation reactions. In few cases, radiation sensitivity can be credited to particular genetic mutations and includes autosomal recessive uncommon diseases like ataxia telangiectasia (AT) [45], AT-like disorder [46], Nijmegen breakage syndrome [47], and radiosensitivity with severe combined immunodeficiency [48]. Heterozygosity for mutations in ATM, the gene mutated in AT, may occur in 1% of individuals and has been reported to confer moderate sensitivity to radiation in tissue culture based studies [49]. In our differentially expressed gene list, we found ATM to be down-regulated (fold change = −2.06843, *p*-value = 1.73 × 10^−7^). A small number of adverse radiation reactions are linked with ATM mutations [50,51,52].

There are some scientific studies hinting towards safe use of alternative medicine like guarana, pineal hormone melatonin, curcumin, amifostine, *etc.* The extract of guarana, a highly caffeinated plant, was used to ease cancer related fatigue at a low cost [53,54]. Evidences had shown pineal hormone melatonin as radioprotective and can be used to reduce the oxidative injuries due to its free hydroxyl radical scavenging capacity [55,56,57]. Curcumin also has a radioprotection effect due to its capability to decrease oxidative stress and inflammatory responses, and also confers radiosensitization perhaps via the upregulation of genes responsible for apoptosis [58]. Multiple studies support the intrarectal application of amifostine (WR-2721/WR-1065), a phosphorothioate during external beam RT for prostate cancer for the prevention of radiation-induced rectal injury [59,60,61].

Radiations are an omnipresent stress to which all life forms are incessantly exposed in the environment. The more we turn towards nuclear power, the greater the concern for accidents, occupational risks or acts of radiological or nuclear terrorism [62]. Presently, the urban population’s exposure to radiation via medical diagnostics and other applications goes beyond their exposure to natural background radiation. Radiation is a double-edged sword: irradiation-induced DNA damage can halt cancer cell proliferation, but collateral radiation damage to adjacent tissues is always a concern. Despite its potential dangers (can even induce tumors and burn skin), the utility of localized radiation in medicine has fueled research and studies focusing on safety. There have been modern advancements in precision technology which lessens the radiation exposure to the healthy tissue, and, therefore, short radiation sessions with escalating doses are feasible for curative local radiation surgery especially in the case of oligometastases [63]. We need drugs (radioprotectors and radiosensitisers) that can protect normal cells while leaving malignant ones susceptible, and ideally, even sensitized to radiation therapy.

## 4. Materials and Methods

### 4.1. Patients and Samples

We retrieved global expression dataset for radiation toxicity studies from NCBI’s GEO database. Dataset were clearly divided into two groups: radiotherapy on cancer patients and radiation studies on healthy individuals or cell lines or mouse models. We focused on actual cancer cases over animal models or cell lines and provided sample information were used to classify case and control. To avoid variations owing to different tissue origins, we included single cancer type and focused on prostate cancer (GSE30174 [5]; GSE69961 [30]) having common platform (GPL570, HG-U133_Plus_2 array chip). To obtain a bigger cohort size for present meta-analysis study, we combined both the prostate cancer studies measuring the transcriptomics level of peripheral blood of patients receiving localized external beam radiation therapy at baseline, midpoints and endpoints.

### 4.2. Gene Expression Analysis

Transcription profiling of a total of 54,675 probe sets targeting ~47,000 gene transcripts in the human genome was done as described previously [64,65]. Partek Genomics Suite version 6.6 (Partek Inc., St. Louis, MO, USA) was used to import affymetrix .CEL files which were normalized using robust multiarray average (RMA) algorithm. Analysis of variance (ANOVA) was applied on the grouped data set to analyze mean expression level on a gene-by-gene basis and list of differentially expressed genes were generated using a Benjamini Hochberg’s false discovery rate (FDR), *p*-value <0.05 with fold change >2 as cut off. Disease and tissue type were two factors in ANOVA model and equal variance were assumed. Spearman’s correlation similarity matrix was used for 2-dimensional unsupervised average linkage hierarchical clustering and classification. Principal component analysis was used to assess overall variance in gene expression and represents cohesive tendency of samples with similar features. Samples clustered tightly together were analyzed and outliers were removed from study to reduce unwanted variance. Venn diagrams were generated to display genes that intersect or non-intersect between groups of differentially expressed genes.

### 4.3. Functional and Pathway Analysis

The principal microarray data analysis was done to detect biological pathways to demonstrate the utilities of more robust biomarker discovery methods for radiation cytotoxicity. Ingenuity Pathways Analysis tool (IPA, build version 338830M, Ingenuity Systems, Redwood City, CA, USA) was used to define biological networks, interaction and functional analysis among the differentially regulated genes in localized external beam radiation treated patients. IPA workflow comprised core and functional analysis and the Ingenuity Knowledge Base was used as a reference data set. Both direct and indirect molecular relationships were included in the analysis settings and significance of relationships was indicated by z-score and Fisher’s exact test *p*-values. Direction and ranking of pathways *i.e.*, activated or inhibited, was determined by the number of uploaded molecules matching a canonical pathway. Network assembly to display significant biological functions was based on the interconnectivity of the uploaded molecules. We uploaded differentially expressed genes along with their *p*-values and fold changes, into the IPA tool for core analysis revealing associated genetic network, canonical pathway, and biofunctions.

### 4.4. Gene Set Enrichment Analysis

A broader understanding of global expression results is possible by grouping the genes of interest into biological processes, cellular component and molecular functions of the genes. Gene ontology (GO) enrichment study was done to functionally classify RT induced significant genes. The implication of this relationship amid transcriptomic data and canonical pathways were calculated by Fisher’s exact test and a cutoff enrichment score >3 (*p*-value < 0.05) was used to identify major overexpression of functional categories.

## 5. Conclusions

Holistic simultaneous measurement of global gene expression using microarray technology transformed the field of cancer biology, and we used it to determine the impact of radiation on cells. We found complex molecular mechanisms including cell cycle arrest, DNA repair and apoptosis involved in regulating the cellular responses to radiation. The most prominent symptom experienced during radiotherapy was fatigue, probably mediated by SNCA and CA1 overexpression, and which may serve as a useful biomarker to understand the mechanisms and pathways related to its development. Hence, an additional impetus for research is the need to develop structure based inhibitors that may have potential co-therapeutic relevance. Also, it is vital to comprehend the radiation dose and mechanisms of response to define an ideal condition where the positive benefits notably outweigh the harmful effects. The cancer patients undergoing RT must be aware of the possible delayed cardiotoxic, hepatotoxic and nephrotoxic effects so that they can adopt a healthy lifestyle and have long term follow up with their health providers. Radiation oncologists should operate on the principle that there is no totally safe radiation dose especially for the heart, and they should avoid direct cardiac radiation and keep the dose as low as possible. Clinicians should look into the deeper aspect of recognizing and advocating radiation-induced organ toxicities and other adverse effects in young adult cancer survivors.

## Figures and Tables

**Table 1:** Top 20 upregulated and differentially expressed genes.

S. No	Gene Symbol	Gene Title	Chromosome Location	Fold-Change	*p*-Value
1	*SNCA*	synuclein, alpha (non A4 component of amyloid precursor)	chr4q21	3.50	2.70 × 10^−6^
2	*CA1*	carbonic anhydrase I	chr8q21.2	3.30	9.12 × 10^−7^
3	*XK*	X-linked Kx blood group	chrXp21.1	2.86	0.000510
4	*GYPB*	glycophorin B (MNS blood group)	chr4q31.21	2.83	1.25 × 10^−6^
5	*HEMGN*	hemogen	chr9q22.33	2.82	4.06 × 10^−5^
6	*GYPA*	glycophorin A (MNS blood group)	chr4q31.21	2.67	1.13 × 10^−5^
7	*BPGM*	2,3-bisphosphoglycerate mutase	chr7q33	2.47	0.002613
8	*FAM46C*	family with sequence similarity 46, member C	chr1p12	2.40	0.002343
9	*ABCC13*	ATP-binding cassette, sub-family C (CFTR/MRP), member 13, pseudogene	chr21q11.2	2.35	1.87 × 10^−5^
10	*FECH*	ferrochelatase	18q21.31	2.33	0.001334
11	*ISCA1*	iron-sulfur cluster assembly 1	chr9q21.33	2.32	1.7 × 10^−6^
12	*CCDC176*	coiled-coil domain containing 176	chr14q24.3	2.29	0.000566
13	*AHSP*	alpha hemoglobin stabilizing protein	chr16p11.2	2.29	7.18 × 10^−7^
14	*YOD1*	YOD1 deubiquitinase	chr1q32.2	2.25	0.000749
15	*NUDT4//NUDT4P1*	nudix (nucleoside diphosphate linked moiety X)-type motif 4	chr12q21//chr1p12-p13	2.19	6.08 × 10^−5^
16	*RHD*	Rh blood group, D antigen	1p36.11	2.18	1.02 × 10^−7^
17	*FGFR1OP2*	FGFR1 oncogene partner 2	chr12p11.23	2.04	0.000354
18	*TSPO2*	translocator protein 2	6p21.1	2.03	6.71 × 10^−13^
19	*ITLN1*	intelectin 1 (galactofuranose binding)	1q23.3	2.02	6.64 × 10^−6^
20	*KRT1*	keratin 1	12q13.13	2.01	0.002745

**Table 2:** Top significant canonical pathways.

Ingenuity Canonical Pathways	-log (*p*-Value)	z-Score	Molecules
Calcium-induced T Lymphocyte Apoptosis	10.8	−3.317	CD247, CD3G, LCK, PRKCQ, CAMK4, TRGV9, ZAP70, NFATC2, HLA-DOB, PRKCH, ITPR1, CD3D, PRKCA
Role of NFAT in Regulation of the Immune Response	10.5	−3.771	CD247, BLNK, FYN, CAMK4, PRKCQ, NFATC3,T RGV9, ITPR1, CD3D, CD3G, LCK, RRAS2, LAT, ZAP70, HLA-DOB, RCAN3, NFATC2, IKBKAP, ATM,ITK
iCOS-iCOSL Signaling in T Helper Cells	10.3	−3.000	CD247, CAMK4, PRKCQ, NFATC3, TRGV9, ITPR1, CD3D, CD3G, LCK, ZAP70, LAT, NFATC2, HLA-DOB, PLEKHA1, ATM,ITK
CD28 Signaling in T Helper Cells	9.61	−3.317	CD247, FYN, CAMK4, PRKCQ, NFATC3, TRGV9, ITPR1, CD3D, CD3G, LCK, ZAP70, LAT, NFATC2, HLA-DOB, ATM,ITK
PKCθ Signaling in T Lymphocytes	8.64	−2.324	CD247,F YN, PRKCQ, NFATC3, TRGV9, MAP3K4, CD3D, CD3G, LCK,RRAS2, ZAP70, LAT, NFATC2, HLA-DOB, ATM
Phospholipase C Signaling	8.01	−3.606	CD247,BLNK, PEBP1, FYN, CAMK4, PRKCQ, NFATC3, TRGV9, ITPR1, CD3D, RHOH, CD3G, LCK, RRAS2, LAT, ZAP70, NFATC2, PRKCH, PRKCA, ITK
Tec Kinase Signaling	5.18	−3.606	FYN, PRKCQ, TRGV9, RHOH, STAT4, BLK, LCK, TXK, TNFRSF25, PRKCH, ITK, PRKCA, ATM
EIF2 Signaling	4.62	−2.828	RPL22, RPS18, RPS4X, RPL10A, RPL14, RRAS2, RPS20, RPL5, RPL36, RPL18, EIF3L, RPS24, ATM
B Cell Receptor Signaling	4.02	−1.897	BLNK, PAX5, ETS1, EBF1, CAMK4, PRKCQ, RRAS2, FOXO1, NFATC3, NFATC2, MAP3K4, ATM
PI3K Signaling in B Lymphocytes	3.88	−2.828	CD81, BLNK, BLK, FYN, CAMK4, RRAS2, NFATC3, NFATC2, PLEKHA1, ITPR1
fMLP Signaling in Neutrophils	3.66	−3.000	CAMK4, PRKCQ, RRAS2, NFATC3, NFATC2, PRKCH, ITPR1, PRKCA, ATM
Apoptosis Signaling	1.56	2.236	PRKCQ, RRAS2, BIRC3, PRKCA, BCL2
Cytotoxic T Lymphocyte-mediated Apoptosis of Target Cells	3.84	1.342	CD247, CD3G, TRGV9, CD3D, BCL2

**Table 3:** Functional annotations and molecules involved in toxicity (cardiotoxicity; hepatotoxicity; nephrotoxicity) resulting from radiation therapy.

Functional Category	Function Annotations	*p*-Value	Molecules
**Cardiotoxicity**			
Cardiac Proliferation	proliferation of cardiomyocytes	1.07 × 10^−1^	FOXP1, NOG
Cardiac Arteriopathy	coronary artery disease	5.09 × 10^−1^	ABCG1, CD47, DOCK9, MARCH6, PDE7A, PRKCH
Cardiac Necrosis/Cell Death	apoptosis of cardiomyocytes and ventricular myocytes	5.36 × 10^−1^	BNIP3, NOG
Heart Failure	chronic heart failure	4.73 × 10^−1^	CA1
Cardiac Infarction	myocardial infarction	1.00 × 10^−1^	CD47, MIAT
**Hepatotoxicity**			
Liver Damage	low and high grade chronic hepatitis C, chronic hepatitis C, hepatotoxicity	1.92 × 10^−2^	CCR7, IMPDH2, RASGRP1
Liver Hyperplasia/Hyper-proliferation	inflammatory hepatocellular adenoma; hepatocellular carcinoma; growth of hepatocellular carcinoma; liver cancer	7.47 × 10^−2^	IL6ST, MYC, + 113 genes
Liver Inflammation/Hepatitis	inflammation of liver; steatohepatitis; chronic hepatitis C	3.26 × 10^−1^	CCR7, IMPDH2, LPIN1, PDE7A
Liver Steatosis	hepatic steatosis; steatohepatitis; nonalcoholic steatohepatitis	1.85 × 10^−1^	LPIN1, PDE7A, RORA
Liver Fibrosis	fibrosis of liver; activation, migration and proliferation of hepatic stellate cells	1.39 × 10^−1^	IL6ST, RORA, CCR7
Liver Necrosis/Cell Death	cell death of liver cells; apoptosis of hepatocytes	3.91 × 10^−1^	BCL2, MYC
Liver Proliferation	proliferation of liver cells; proliferation of hepatocytes; proliferation of hepatic stellate cells	2.24 × 10^−1^	IL6ST, LY9, MYC
**Nephrotoxicity**			
Renal Necrosis/Cell Death	apoptosis of kidney cell lines; apoptosis of podocytes; cell death of kidney cell lines; cell viability of kidney cell lines	5.51 × 10^−2^	AQP3,AAK1,BCL2, BIRC3, BNIP3, DDX17, FOXO1, ITPR1, MYC, PRKCA, SNCA, TNFRSF25
Nephrosis	nephrosis; minimal change nephrotic syndrome; autosomal recessive steroid-resistant nephrotic syndrome; steroid dependent nephrotic syndrome	2.43 × 10^−1^	IMPDH2, MS4A1
Renal Nephritis	IgA nephropathy; membranous glomerulonephritis; lupus nephritis	1.13 × 10^−1^	IMPDH2, MS4A1
Renal Proliferation	proliferation of mesangial cells; proliferation of kidney cell lines	3.96 × 10^−1^	CCR7, HSP90AB1, KMT2A, SFPQ
Kidney Failure	end stage renal disease	4.63 × 10^−1^	IMPDH2, PDE7A

## Supplementary Materials

Supplementary materials can be found at http://www.mdpi.com/1422-0067/17/2/250/s1.

## Abbreviations

GO: Gene ontology
RT: Radiation therapy
CA1: carbonic anhydrase I
SNCA: a-synuclein
APCs: antigen-presenting cells
CRF: cancer related fatigue
MS4A1: membrane-spanning 4-domains, subfamily A, member 1
