# Supplementary material for: Assessment of Radiation Induced Therapeutic Effect and Cytotoxicity in Cancer Patients Based on Transcriptomic Profiling
Source: Int J Mol Sci. 2016 Feb 19;17(2):250. doi: 10.3390/ijms17020250 (PMC4783980; doi:10.3390/ijms17020250)
Supplement: Supplementary file 1 [file ijms-17-00250-s001.zip › ijms-116997-Supplementary Materials/ijms-116997-Supplementary Table S3.pdf]

Supplementary Materials: Assessment of Radiation Induced Therapeutic Effect and Cytotoxicity in Cancer Patients Based on Transcriptomic Profiling

Sajjad Karim, Zeenat Mirza, Adeel G. Chaudhary, Adel M. Abuzenadah, Mamdooh Gari and Mohammed H. Al-Qahtani

Table S3. Top functional annotations identified by GO analysis.

GO ID	Function	Enrichment Score	Enrichment *p*-Value	% Genes in Group That Are Present
2376	Immune system process	36.9101	9.34×10^{-17}	4.83696
46649	Lymphocyte activation	36.6413	1.22×10^{-16}	10.8025
45321	Leukocyte activation	35.9238	2.5×10^{-16}	9.64467
2682	Regulation of immune system process	32.6924	6.34×10^{-15}	5.03018
30217	T cell differentiation	32.5937	6.99×10^{-15}	19.4175
2694	Regulation of leukocyte activation	31.9539	1.33×10^{-14}	8.53933
50865	Regulation of cell activation	31.3614	2.4×10^{-14}	8.1761
51249	Regulation of lymphocyte activation	31.2634	2.65×10^{-14}	9.06736
42110	T cell activation	30.6735	4.77×10^{-14}	12.381
70489	T cell aggregation	30.6735	4.77×10^{-14}	12.381
71593	Lymphocyte aggregation	30.4459	5.99×10^{-14}	12.2642
70486	Leukocyte aggregation	29.7789	1.17×10^{-13}	11.9266
1775	Cell activation	29.4058	1.7×10^{-13}	7.06076
2696	Positive regulation of leukocyte activation	28.4235	4.53×10^{-13}	9.96564
50867	Positive regulation of cell activation	27.6516	9.8×10^{-13}	9.66667
2684	Positive regulation of immune system process	27.3908	1.27×10^{-12}	5.83524
30098	Lymphocyte differentiation	27.2383	1.48×10^{-12}	12.3656
51251	Positive regulation of lymphocyte activation	27.0592	1.77×10^{-12}	10.1887
7159	Leukocyte cell-cell adhesion	26.5689	2.89×10^{-12}	10.4
6955	Immune response	25.6371	7.34×10^{-12}	4.89731
34109	Homotypic cell-cell adhesion	24.897	1.54×10^{-11}	9.66543
50851	Antigen receptor-mediated signaling pathway	24.7816	1.73×10^{-11}	11.5789
2768	Immune response-regulating cell surface receptor signaling pathway	23.4633	6.46×10^{-11}	7.08333
2521	Leukocyte differentiation	23.458	6.49×10^{-11}	9.43396
2429	Immune response-activating cell surface receptor signaling pathway	22.838	1.21×10^{-10}	8.81356
2764	Immune response-regulating signaling pathway	21.1685	6.41×10^{-10}	6.21762
46632	Alpha-beta T cell differentiation	21.015	7.47×10^{-10}	28.5714
50776	Regulation of immune response	20.606	1.12×10^{-9}	5.01567
34110	Regulation of homotypic cell-cell adhesion	19.2966	4.16×10^{-9}	8.02676
2253	Activation of immune response	19.2894	4.2×10^{-9}	6.63717
2757	Immune response-activating signal transduction	18.9626	5.82×10^{-9}	6.91358
50863	Regulation of T cell activation	18.914	6.11×10^{-9}	8.18505
46631	Alpha-beta T cell activation	18.5652	8.65×10^{-9}	22.7273
1903037	Regulation of leukocyte cell-cell adhesion	18.3818	1.04×10^{-8}	7.95848
50778	Positive regulation of immune response	17.9194	1.65×10^{-8}	5.71429
50853	B cell receptor signaling pathway	17.6644	2.13×10^{-8}	20.8333
2250	Adaptive immune response	17.5544	2.38×10^{-8}	9.62567
50870	Positive regulation of T cell activation	16.9042	4.56×10^{-8}	9.23077
48583	Regulation of response to stimulus	16.6195	6.06×10^{-8}	3.10453
22407	Regulation of cell-cell adhesion	16.6074	6.13×10^{-8}	6.75676
34112	Positive regulation of homotypic cell-cell adhesion	16.5917	6.23×10^{-8}	9.04523
1903039	Positive regulation of leukocyte cell-cell adhesion	16.5149	6.72×10^{-8}	9
22409	Positive regulation of cell-cell adhesion	15.8545	1.3×10^{-7}	8.18966
42101	T cell receptor complex	15.362	2.13×10^{-7}	40
50852	T cell receptor signaling pathway	15.3487	2.16×10^{-7}	10

Table S3. *Cont.*

GO ID	Function	Enrichment Score	Enrichment *p*-Value	% Genes in Group That Are Present
16337	Single organismal cell-cell adhesion	15.0338	2.96×10^{-7}	6.04651
48584	Positive regulation of response to stimulus	14.9962	3.07×10^{-7}	3.56037
45785	Positive regulation of cell adhesion	14.1967	6.83×10^{-7}	6.31868
45058	T cell selection	14.0637	7.80×10^{-7}	25
98602	Single organism cell adhesion	13.9996	8.32×10^{-7}	5.72687
